# Effects of Electromagnets on Bovine Corneal Endothelial Cells Treated with Dendrimer Functionalized Magnetic Nanoparticles

**DOI:** 10.3390/polym13193306

**Published:** 2021-09-28

**Authors:** Shadie Hatamie, Po-Jen Shih, Bo-Wei Chen, Hua-Ju Shih, I-Jong Wang, Tai-Horng Young, Da-Jeng Yao

**Affiliations:** 1 College of Medicine, National Taiwan University, Taipei 10048, Taiwan; ijong@ntu.edu.tw; 2 Department of Biomedical Engineering, National Taiwan University, Taipei 10617, Taiwan; thyoung@ntu.edu.tw; 3 Institute of Nanoengineering and Microsystem, National Tsing Hua University, Hsinchu 30013, Taiwan; s106035802@m106.nthu.edu.tw (B.-W.C.); djyao@mx.nthu.edu.tw (D.-J.Y.); 4 Institute of Applied Mechanics, National Taiwan University, Taipei 10617, Taiwan; d04543001@ntu.edu.tw

**Keywords:** SPION, G4-dendrimer, magnetic device BCECs, cell migration, MRI

## Abstract

To improve bovine corneal endothelial cell (BCEC) migration, enhance cell energy, and facilitate symmetric cell distribution in corneal surfaces, an electromagnet device was fabricated. Twenty nanometer superparamagnetic iron oxide nanoparticles (SPIONs) functionalized with fourth-generation dendrimer macromolecules were synthesized, and their size and structure were evaluated using transmission electron microscopy (TEM), X-ray photoelectron spectroscopy (XPS), and Fourier transform infrared spectroscopy (FTIR). The results confirmed the configuration of the dendrimer on the SPION surfaces. In vitro biocompatibility was assessed using the 3-[4,5-dimethylthiazol-2yl]-2,5-diphenyl tetrazolium bromide assay. No significant toxicity was noted on BCECs within 24 h of incubation. In the cell migration assay, cells treated with dendrimer-coated SPIONs exhibited a relatively high wound healing rate under sample addition (1 μg/mL) under a magnetic field. Real-time PCR on BCECs treated with dendrimer-coated SPIONs revealed upregulation of specific genes, including AT1P1 and NCAM1, for BCECs-dendrimer-coated SPIONs under a magnetic field. The three-dimensional dispersion of BCECs containing dendrimer-coated SPIONs under a magnetic field was evaluated using COMSOL Multiphysics software. The results revealed the BCECs-SPION vortex pattern layers in the corneal surface corresponded to the electromagnet’s displacement from the ocular surface. Magnetic resonance imaging (MRI) indicated that dendrimer-coated SPIONs can be used as a T_2_ contrast agent.

## 1. Introduction

The use of magnetic nanoparticles (MNPs) coupled with magnetic base devices has been increasing in the biomedical field [1,2,3,4]. Among them, superparamagnetic iron oxide nanoparticles (SPIONs) are widely used in nanomedicine for diagnoses, therapeutics, drug, and gene delivery systems [5,6,7,8]. To enhance SPION biocompatibility, solubility, and stability in physiological conditions, surface modifications with biocompatible polymers are necessary. Surface coating of SPIONs by dendrimers as three-dimensional (3D) hydrophilic polymers with highly branched macromolecular structures can increase their solubility in water by reducing their intermolecular attractions through an increase in steric hindrance [9,10]. Spatial structures of dendrimer-functionalized SPIONs (D-SPIONs), which are useful in conjugation with other chemicals and for encapsulation of drugs, serve as excellent imaging probes [11,12], such as for MRI, due to their prolonged half-life [13]. Moreover, the high colloidal stability and excellent biocompatibility of D-SPIONs are critical properties for MRI contrast media [14,15].

Recently, magnetic nanoparticle applications in ocular systems, such as corneal endothelial cell (CEC) migration and proliferation [16,17] under external magnetic forces, have received scholarly attention. In relevant studies, the CECs’ viability and morphology remained intact [16,18], and the cells’ metabolic activity was enhanced [19,20]. SPION-labeled CEC densities can be increased by using permanent magnets of different shapes and sizes. However, establishing a uniform distribution of CECs on the corneal surface remains challenging. We synthesized 20 nm SPIONs by using chemical decomposition methods. Four generations of dendrimers, namely poly(amidoamine) (PAMAM), were synthesized to functionalize SPIONs through divergent and convergent methods as stepwise chemical approaches. Functionalized SPIONs with dendrimers possess multiple amine groups in their surfaces for active connections involving biomolecule attachment. D-SPIONs can be used as MRI contrast media inside the ocular system for bioimaging. Furthermore, BCECs were exposed by D-SPIONs and manipulated under the tapping mode of two vertical electromagnets facing each other. Real time polymerase chain reaction was performed to assess the expression of genes in the BCECs treated with D-SPIONs, both under and not under a magnetic field. The simulation study confirmed that the uniform distribution of the BCECs under magnetic fields corresponds to the electromagnet’s displacement from the ocular surface. We believe that the interfacing between the magnetic fields and D-SPION-tagged BCECs can be further used as effective therapeutic tools for the ocular system.

## 2. Materials and Methods

### 2.1. Materials

Iron (III) acetylacetonate (Fe(acac)_3_), oleic acid, oleylamine, octyl ether, methyl acrylate, ethylenediamine, PBS, and dimethyl sulfoxide were purchased from Sigma Aldrich (Sigma Aldrich, St. Louis, MO, USA), and 3-aminopropyl triethoxysilane (APTES) was purchased from Merck (Merck & Co., Inc., Kenilworth, NJ, USA). PBS, and dimethyl sulfoxide.

#### 2.1.1. Synthesis of SPIONs

The SPIONs were synthesized by thermal decomposition of the Fe(acac)_3_ inside a solution containing oleic acid, oleylamine, and octyl ether. The mixture was stirred for 30 min at room temperature under a nitrogen flow. Next, the temperature was increased to 300 °C for 1 h until the Fe(acac)_3_ had been completely decomposed. Finally, the solution was cooled to room temperature, washed several times with ethanol, and collected using permanent magnets [21].

#### 2.1.2. D-SPIONs

**Figure 1:**
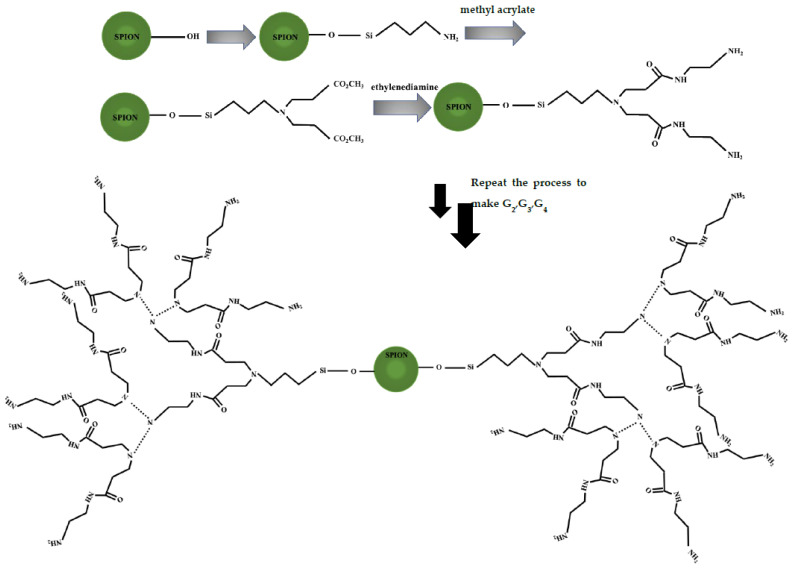
Process of fourth-generation dendrimer functionalization of the SPION surfaces.

The PAMAM-functionalized SPIONs were fabricated through three reaction steps, as described previously [22]. During the fabrication process, SPION surfaces should first be modified by amine group attachment for subsequent dendrimer coating. In brief, APTES in ethanol (10 mL, 10% vol/vol) was added dropwise to the SPIONs dispersed in ethanol through sonication. The mixture was stirred for 7 h, separated by magnets, washed with ethanol five times, and collected for further analysis. For the synthesis of the PAMAM-coated SPIONs, the methyl acrylate (15 mL, 20% vol/vol) was dissolved in methanol and added to the SPIONs functionalized with aminosilane (20 mL, 5% wt/vol) and stirred for 48 h. This was followed by 2 h of sonication. Next, the SPIONs were washed five times. Finally, ethylenediamine was dissolved in methanol (10 mL, 50% vol/vol), and added to the modified SPIONs, and the mixture was sonicated for 3 h. The resulting first-generation dendrimer on the SPION surface was then subjected to washing and purification using magnets. The two main reactions, involving the generation of ester bonds through the connection of methyl acrylate and amino silane in the first step and followed by the amidation of ester bonds with ethylenediamine in the second step, was repeated three times to fabricate the fourth-generation dendrimer branches (Figure 1).

### 2.2. Characterization Techniques

#### 2.2.1. TEM, FTIR, XPS, Dynamic Light Scattering (DLS), and Zeta Potential

TEM was performed under an accelerating voltage of 200 kV. A field-emission Tecnai F20 electron gun of ZrO/W (100) Schottky type (resolution ≤0.23 nm) was used on a (Philips/FEI Corporation, Eindhoven, The Netherlands) TEM system. XPS was performed using an Al Kα X-ray source under a working energy of 1486.6 eV in a vacuum of approximately 10^−7^ Pa (XPS, ESCA000600). FTIR spectroscopy was conducted by grinding the SPIONs and D-SPIONs into powder with the KBr to make pellets using a Perkin Elmer system (PerkinElmer, Inc. Waltham, MA 02451 USA) in the range of 400–4000 cm^−1^. The DLS and zeta potential of the SPIONs and D-SPIONs dispersed in deionized water were measured using Malvern instrument (Malvern Panalytical Ltd, Great Malvern, UK).

#### 2.2.2. Bovine Corneal Endothelial Cell Preparation

The bovine eyes were obtained from a local butchery and fumigated using an iodine solution [23]. After the eyes were washed with phosphate-buffered saline (PBS), the corneas were removed, as were the Descemet membranes. Subsequently, the Descemet membranes were incubated with trypsin under a CO_2_ atmosphere at 37 °C for 45 min until the cells were detached. After centrifugation at 1000 rpm for 5 min, the BCECs were collected and cultured.

#### 2.2.3. MTT Cell Viability Assay

The 3-[4,5-dimethylthiazol-2yl]-2,5-diphenyl tetrazolium bromide (MTT) (Sigma Aldrich, St. Louis, MO, USA). viability assay of D-SPIONs was performed using bovine CECs (BCECs) for the duration of 24, 48, and 72 h. For the MTT assay, the 6 × 10^3^ cells/well are cultured and grown on 96-well plates at 37 °C in an endothelial cell medium and 1% penicillin for 24 h. The cells were then incubated with five concentrations of D-SPIONs (10, 30, 50, 70, and 100 µg/mL) for 24, 48, and 72 h. At the end of the incubation periods, 10 μL of MTT was added to each well, and the cells were kept in the incubator for 3 h. Next, the cells were washed with PBS, 100 μL of dimethyl sulfoxide was added to each well, and the plate was shaken for 10 min. Finally, the optical density of each cell was determined at 595 nm on a microplate reader (Bio-Rad S/N 21648, Pleasanton, CA, USA) Using the absorbance data, the percentage of live cells was calculated [24,25].

#### 2.2.4. MRI of Fourth-Generation D-SPIONs

To evaluate the MRI relaxivity of the fourth-generation D-SPIONs as T_2_-weighted contrast agents, a 7 Tesla Bruker BioSpec MRI system (Bruker BioS pec 70/30 US, Billerica, MA, USA) was used. T_2_-weighted relaxation times were defined by a multiecho spin-echo sequence (repetition time: 4000 ms; echo time:18 ms). The r_2_ relaxivity was collected from the slope of 1/T_2_ versus the D-SPION concentrations, and signal decay data were collected. Phantoms were prepared in varying concentrations (0.1, 0.2, 0.3, 0.5, 0.7, and 1 mg/mL) by adding 1% agarose. The samples were then transferred to 0.5 mL microtubes and placed inside a 7 cm coil. Images were taken using the designed sequences with a matrix size of 128 × 128, a field of view of 6 × 6 cm^2^, and a slice thickness of 1 mm.

#### 2.2.5. Electromagnet Setup

The electromagnet device consisted of two electromagnets facing each other under an up/down adjustable magnetic field gradient. The magnets were placed under a step functional magnetic field of approximately 500 G every 2 s and were triggered by using the Arduino device, delivering a step-functional ± 5 volts. The magnetic field consisted of two commercial electromagnets (Grove, seeed studio, Shenzhen, China) which were made by a thousand-rounds wire and a cold-rolled steel rod, and the working gap was tunable. The strength of the magnetic field was measured using a Tesla meter (TM-801 EXP, KANETEC) and was sensed at the bottom position of the dish [26].

After the BCECs were cultured and the number of cells reached the experimental requirements (10^4^ BCECs in each 96-well plate), D-SPIONs (10 µg/mL) were added to the dish containing the cells and incubated for 24 h such that they could be absorbed by the cells. Finally, the BCECs were washed with PBS, and the D-SPION cells were placed under an applied magnetic field in an incubator for 1 month (Figure 6a).

#### 2.2.6. BCEC Migration Assay

An in vitro BCEC scratch assay was performed. In brief, BCECs were seeded in a 96-well plate at a density of 10^4^ cells/well and incubated for 24 h. Next, two concentrations of dendrimer coated iron oxide (1 and 10 μg/mL) were added to each well and incubated for 24 h at 37 °C under a CO_2_ atmosphere (untreated BCECs were used as controls). Next, straight lines are scratched in each well using a sterile 100 μL pipette tip. Images of the scratches in each well were examined at 100 × magnification under an inverted microscope and analyzed with ImageJ software. Each experiment was performed twice. The scratch experiments were repeated for the cell migration assay for the same concentrations under a magnetic field produced by electromagnets in tapping modes. 

#### 2.2.7. Simulation Study 

A 3D simulation study was performed by COMSOL (COMSOL, Inc. Burlington, MA, USA), to confirm the distribution of the BCECs holding nanoparticles on the ocular surface. The uniform distribution of the CECs on the ocular surface is challenging for gene delivery and drug delivery toward the cornea. The delivery to CECs containing SPIONs was determined to be nonuniform, creating cell accumulations on the ocular surface. Herein, the density of the D-SPIONs was less than 10^13^ (1/m^3^); therefore, their contribution was ignored in the simulation. According to Maxwell’s equation, the magnetic flux density of the magnet can be expressed as:

(1) $$B_{magnet}=\mu_{0}(H+M)$$

where the *μ*_0_ denotes permeability, *H* is the magnetic field intensity, and *M* represents the magnetization.

As presented in Figure 7, the cylindrical permanent magnet radius is labeled as *r_m_*, the distance between the magnet and the eyeball was denoted as *D*, and the eyeball radius was denoted as *r*. Physical properties of each item used in simulation are listed in Table 1.

#### 2.2.8. Real-Time PCR Assay

Real-time PCR was performed according to the manufacturer’s protocol. The cDNA was synthesized using RNA, a random hexamer primer, Revert Aid Reverse Transcriptase, and a dNTP mix. The cDNA of the treated and untreated D-SPION cells were diluted using deionized water.

Real-time PCR was conducted using the Applied Biosystems 7500 Real-Time PCR System (Applied Biosystems, Foster City, CA, USA). The 20 μL container was filled with cDNA and the as prepared solution contained SYBR Green Real-Time PCR Master Mix, gene-specific primers, and deionized water. The gene-specific primers are listed in Table 2. Glyceraldehyde 3-phosphate dehydrogenase (*GAPDH*) was used to normalize the gene expression values, and melt curve analysis was conducted to assess the reaction specifications [27,28]. The 2^−ΔΔCt^ method was employed to analyze the data. The cycle thresholds for the intrinsic control of *GAPDH* were derived from the cycle thresholds for all the cell markers. The fold changes for the D-SPION cells under a magnetic field were normalized to those of the untreated cells, and the values underwent an exponential linear conversion by using the 2^−ΔΔCt^ equation [29].

#### 2.2.9. Statistical Analysis

Analyses were conducted using SPSS software, and one-way analysis of variance was performed to find differences between SPIONs and D-SPIONs. A *p*-value of ≤0.05 was considered significant. Data are presented as ± standard errors of the mean.

## 3. Results and Discussion

### 3.1. Shape, Size, Surface Charge, and Structural Bonding of D-SPIONs

**Figure 2:**
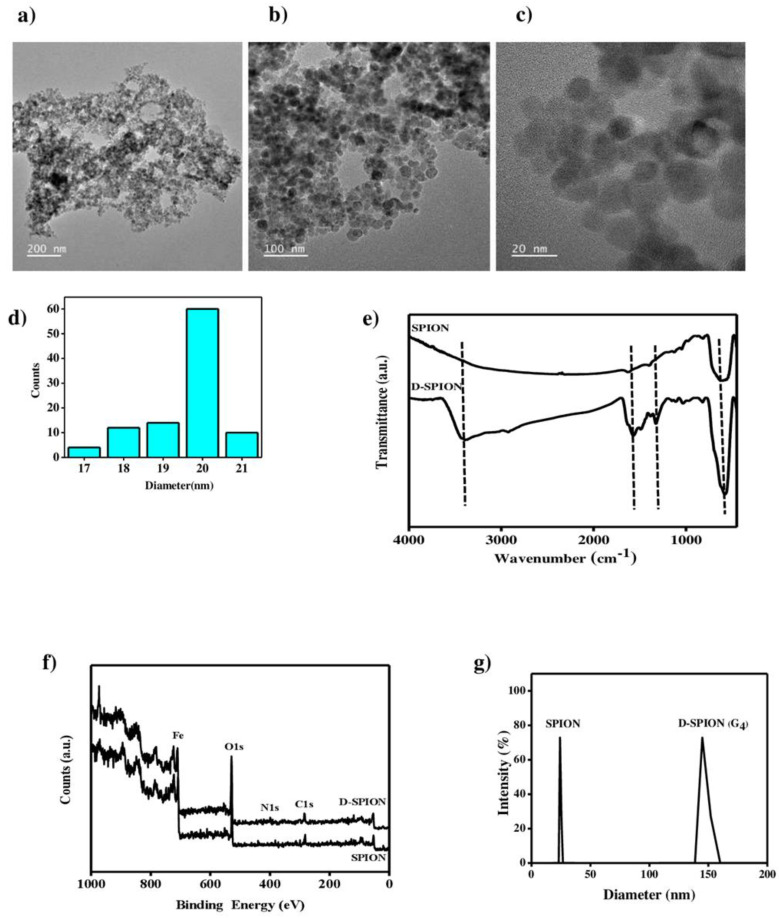
Shows the (**a**–**c**) TEM images of the D-SPIONs; (**d**) size distribution histogram of D-SPIONs; (**e**) FTIR spectra; (**f**) XPS survey patterns; (**g**) DLS of the SPIONs and D-SPIONs.

The shape, size, Surface charge, and structural bonding of D-SPIONs are shown in Figure 2.

#### 3.1.1. TEM

TEM images of the D-SPIONs are presented in Figure 2a–c. The D-SPIONs were almost spherical and had a uniform size distribution. The D-SPIONs’ average size, as measured by applying ImageJ software to the TEM images, was approximately 20 ± 3 nm (Figure 2d).

#### 3.1.2. FTIR

The attachment of the fourth-generation dendrimers on SPION surfaces was confirmed using Fourier transform infrared (FTIR) spectroscopy. As presented in Figure 2e, the sharp peak at 590 cm^−^^1^ was assigned to the Fe–O stretching vibration of the iron oxide nanoparticles at their tetrahedral site bond. As presented in the D-SPION spectra, the peaks at 1489 and 1567 cm^−1^ were attributed to the –CO–NH– groups, and the peak at 1023 cm^−1^ was ascribed to the –CO–NH_2_ groups, which are highly electronegative. Both peaks confirm the existence of the dendrimer branch on the SPION surfaces. Moreover, the peak at 3409 cm^−1^ was related to the –NH_2_-group vibration of the dendrimer [30]. Overall, the FTIR spectra indicate that the dendrimer modified the SPIONs.

#### 3.1.3. XPS

Surface analysis of the SPIONs and D-SPIONs (G_4_) was performed using XPS (Figure 2f). The peak in the SPION spectra corresponded to the C1s (285 eV), O1s (528.5 eV), and Fe2p (710.3 eV). In the D-SPION spectra, a peak corresponding to the nitrogen sign at N1s (398.2 eV) was observed [2]. XPS analysis revealed the presence of the dendrimer on the iron oxide nanoparticle surfaces. The peak position of N1s, which appeared at 390–400 eV, confirmed that the dendrimer molecules were attached to the iron oxide surfaces [31]. The SPION peak Fe2p and the oxygen peak O1s exhibit a reduction in their intensity by covering the SPIONs with the PAMAM layers, but the amount of the carbon (C1s) atoms is increased, and the nitrogen peaks appear in the D-SPION spectra. The increase in dendrimer layers at the SPION surface reduces the amount of the iron and oxygen molecules through a process wherein the dendrimer thickness is increased [32].

#### 3.1.4. DLS and Zeta Potential 

The hydrodynamic size of SPIONs and the fourth-generation D-SPIONs were measured using DLS. As shown in Figure 2g, the hydrodynamic size of the SPIONs was increased after they were coated with the dendrimer. The hydrodynamic diameter of the SPIONs and D-SPIONs exceed the actual sizes seen in the TEM images; this inaccuracy is the main disadvantage of using DLS. In DLS, signals from larger particles can suppress signals from smaller particles, and it could be associated with the higher contribution of the light scattering to the nanoparticle size distributions. This means that higher size distributions can lead to errors in DLS size measurements, yielding discrepancies with the real size indicated in TEM. Furthermore, for highly magnetic nanoparticles, nanoparticle agglomeration occurs during measurement, which can affect the analytical signals. The surface charge study of SPIONs and D-SPIONs, as performed through zeta potential measurement, was −24 and −0.6 mV, respectively. These results can be attributed to the carboxylate moiety on the SPION surface. This surface is further functionalized by the amine groups of PAMAM producing D-SPIONs [33].

### 3.2. MTT Cytotoxicity Assay

**Figure 3:**
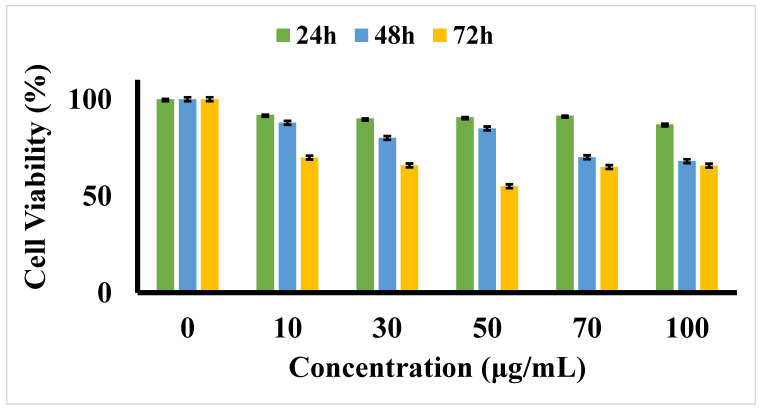
Cell viability MTT assay for varying concentrations of D-SPIONs on BCECs over 24, 48, and 72 h. Data are presented as means ± standard deviations (n = 3). One-way analysis of variance and the Tukey post hoc test revealed no significant difference based on the different incubation times (*p* > 0.05).

The 3-[4,5-dimethylthiazol-2yl]-2,5-diphenyl tetrazolium bromide (MTT) cytotoxicity assay to investigate the cytotoxicity of five concentrations of D-SPIONs under 24, 48, and 72 h of treatment on BCECs (Figure 3). The BCECs exhibited various biological responses when exposed to differing dosages of D-SPIONs over varying durations. No significant toxicity of D-SPIONs on BCECs was noted up to 100 μg/mL for 24 h. The cell viability for the 100 μg/mL under 48 and 72 h of incubation was 68% and 65%, respectively. Because iron oxide nanoparticles are biocompatible and nontoxic, they display slight cytotoxicity against BCECs [28,34].

### 3.3. MRI of D-SPIONs

**Figure 4:**
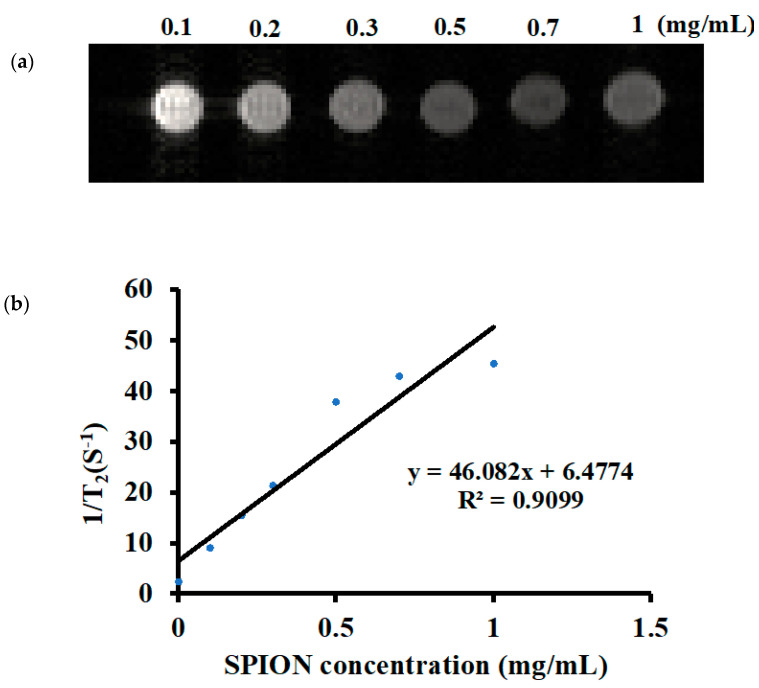
(**a**) T_2_-weighted magnetic resonance images at six D-SPION concentrations; (**b**) linear fitting of relaxation rate (1/T_2_) versus D-SPION concentrations. The relaxivity values of r_2_ were calculated from the slope of the graph.

Figure 4 presents the MRI images of the D-SPIONs specifically, T_2_ images at various sample concentrations (Figure 4a) and relaxation rates against various sample concentrations (Figure 4b). Dose dependence was noted, as indicated by the darkening that occurred as the spin–spin relaxation time (T_2_) was shortened. The transverse relaxation rate (1/T_2_) versus the various concentrations of the D-SPIONs is presented in a linear plot [35]. The relaxivity r_2_ was calculated to be 46 mM^−^^1^S^−1^.The SPIONs and D-SPIONs were reported to enhance the transverse relaxation rate more than the longitudinal relaxation rate and be a negative contrast agent. Dendrimer-based MRI contrast agents has also been used for imaging brain tumors [36]. As synthetic macromolecules, dendrimers can improve the relaxivity in MRI because of their 3D structures, tunable sizes, and abundant surface terminals. Furthermore, they can increase the positivity of iron oxide. Coating dendrimers on the SPION surface promote their specific targeting, enhance MRI image quality, reduce their toxicity on cells, and allow for safe in vivo imaging.

### 3.4. Cell Migration Assay under Magnetic Fields

**Figure 5:**
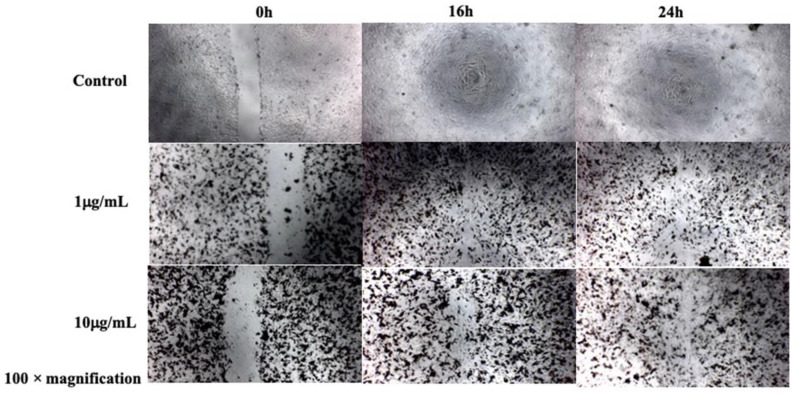
Migration assay of BCECs treated under varying concentrations of D-SPIONs over 16 and 24 h.

**Figure 6:**
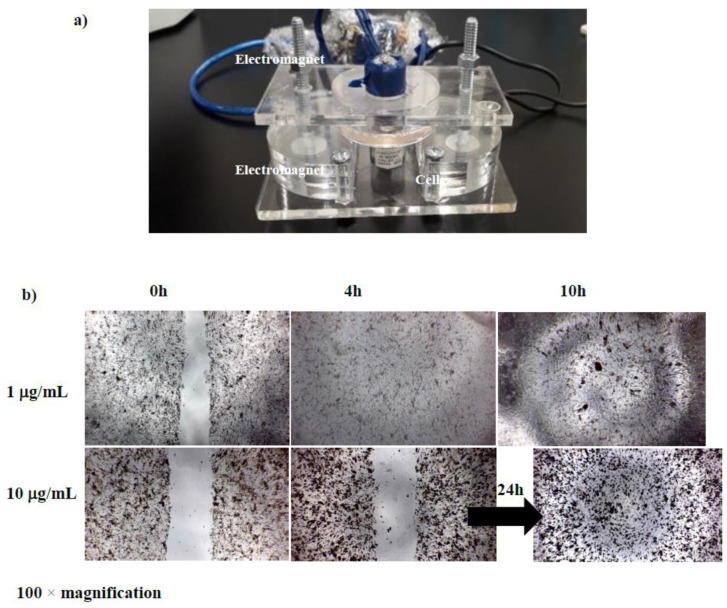
(**a**) The magnetic device used in this experiment; (**b**) cell migration assay of BCECs containing different concentrations of D-SPIONs under a gradient magnetic field.

Migration in CECs is an essential step in the wound healing process. Cell migration was suppressed in the BCECs treated with D-SPIONs compared with in the control cells (BCECs without nanoparticles). Moreover, the migration of the BCECs containing 1 μg/mL D-SPIONs was faster than that of the cells containing 10 μg/mL SPIONs (Figure 5). Increases in nanoparticle agglomeration, cell uptake capacities, and nanoparticle moieties on the cell surfaces are the main reasons for the delay in cell migration under increases in the D-SPION concentration [37]. Under a magnetic field produced by electromagnets in tapping mode, wound closure in the BCECs treated with 1 μg/mL SPIONs took 10 h. This is even faster than wound closure in the control BCECs. Notably, the magnetic vortex patterns were created by BCECs containing D-SPIONs by applying the magnetic field for both concentrations of D-SPIONs (1 and 10 μg/mL) at different times (Figure 6b) [38].

### 3.5. COMSOL Multiphysics Simulation Study

**Figure 7:**
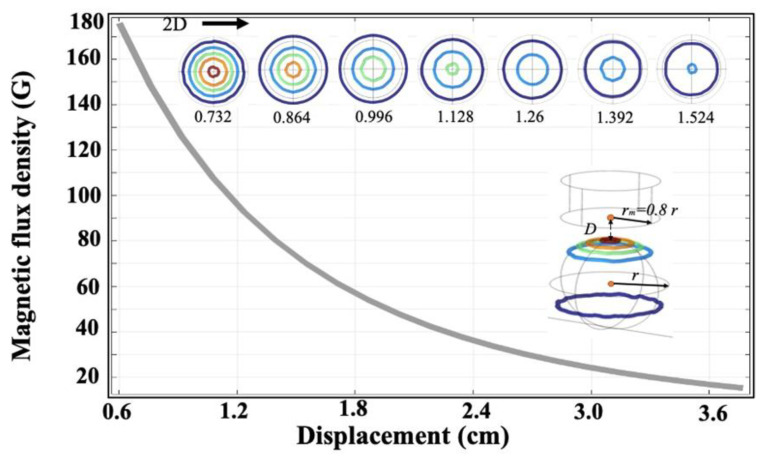
Magnetic flux density versus the electromagnet’s displacement from the ocular surface.

In the simulation, we assumed no current conveyance in the domain (H = 0); next, the magnetic flux density was adjusted through the magnet. The magnetization was calculated to be 10^4^ (A/m) in the *z* direction from the electromagnet under a *r_m_* of *0.8r* and a *D* of *0.2r*. Table 1 lists the other parameters. The magnetic flux density created on the ocular surface was inversely proportional to the magnet’s displacement from the ocular surface (Figure 7). When the displacement was increased, the magnetic flux density decreased, affecting the number of magnetic patterns on the ocular surface. In this regard, the isometric line vortex around the magnetization direction was considered.

### 3.6. Real-Time PCR

**Figure 8:**
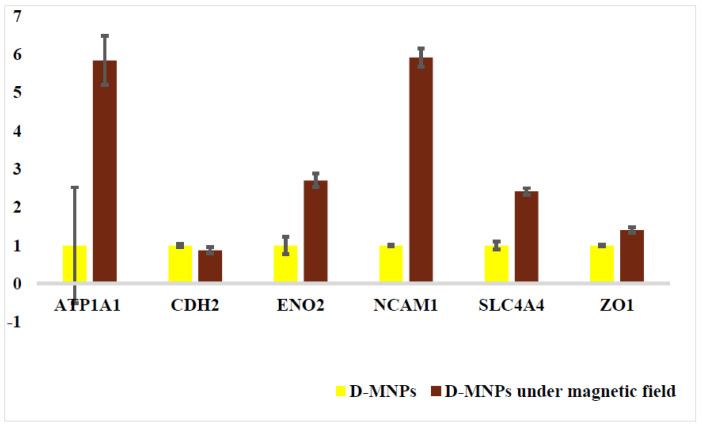
Gene expression level of BCECs incubated with D-SPIONs and treated with an electromagnet system using real time PCR. The genes were normalized using GAPDH. The cells incubated with the MNPs were used as the control group and statistically differences were (*p* < 0.05; n = 3). Data are presented as the means standard deviations of three experiments.

To investigate the change in the cellular identity of the BCECs before and after the treatments with the alternative magnetic field, real-time PCR was conducted (Figure 8). To measure the mRNA expression of specific genes, BCEC-specific markers (ATP1A1, CDH2, ENO2, NCAM1, SLC4A4, and zonula occludens-1 [ZO-1]), were used for untreated cells and treated cells for 1 month [39,40]. The markers were selected based on strong expression of genes in BCECs, and low expression of them in stromal cells. Comparison of the gene expression of D-SPION endothelial cells before and after 1-month cell treatment revealed an approximate sixfold growth in ATP1A1 and NCAM1 expression and an approximate 2.5-fold growth in ENO2 and SLC4A4. No major changes were observed in ZO-1 and CDH_2_ expression; moreover, no clear differences in the tight junction morphology of the BCECs before and after the treatments (ZO-1 is a protein involved in cellular function). The significantly high increase in adenine triphosphatase (ATPase)Na+/K+ transporting α1polypeptide (ATP1A1) was reported. ATP1A1 supplies instructions for fabricating the alpha-1 subunit of a Na^+^/K^+^ ATPase, which is known as a protein pump. ATP is then used by the protein to transport K^+^ ions into and Na^+^ ions out of cells [41]. The solute carrier 4 SLC4 transporters (except SLC4A11) are divided into three groups based on their functions: anion exchangers, sodium bicarbonate cotransporters (NBCs), and sodium-driven Cl–/HCO– exchangers [42,43]. Next, the expression of SLC4A4 as an electrogenic NBC1 was localized in the basolateral membrane of corneal epithelial cells. The expression of the SLC4A4 gene (as a primary member in the corneal endothelium) suggests that NBCs are the main bicarbonate transporters in the corneal endothelium. Moreover, for the SLC4A4 gene upregulated, the increase of salt content in the medium after magnetic treatments could increase the expression of solute transporter [44]. The expression of the neural cell adhesion molecule, which is encoded by the NCM1 gene, was significantly increased. NCM, an adhesion molecule known as the immunoglobulin gene [45], can interface neuron-muscular and neuron–neuron adhesion through their interactions and initiate cell matrix interactions. NCAM expression by HCECs has a neural crest origin. NCAM overexpression might correspond to the adhesion of the BCECs to each other.

## 4. Conclusions

The SPION surfaces were functionalized with the fourth-generation dendrimer for better spatial distance. The TEM images revealed that the D-SPIONs measured approximately 20 nm. FTIR spectroscopy and XPS confirmed the coating of the dendrimer on the SPION surfaces. The zeta potential of the SPIONs changed from approximately −28 to −0.6 mV after dendrimer functionalization because of the induction of amine groups on its surfaces. The MTT assay indicated that the D-SPION had low toxicity on the BCECs over 24 h. Moreover, the coated nanoparticles could be used as contrast agents for in vivo bioimaging. The cell migration assay showed fast migration within 10 h of treatment with 1 μg/mL D-SPIONs under magnetic fields, as well as a uniform distribution of BCEC-containing D-SPIONs. These vortex pattern of BCECs containing D-SPIONs was simulated using COMSOL Multiphysics software for in vivo applications. The real time-PCR results for BCECs treated with D-SPIONs under magnetic fields indicated the overexpression of the AT1P1 and NCAM1 genes, which could explain the rapid wound closure in migration assay.

## Figures and Tables

**Table 1:** Physical properties of each item included in the simulation.

Items	Electrical Conductivity	Relative Permittivity	Relative Permeability
Eye ball	0.05	83	0.999992
Air	0.01	1	1.00000037
Magnet	1	1	

**Table 2:** Primers used in this study.

Gene	Gene Sequence
ATP1A1 FW	5′-CAAGCCCTCGTGATTCGAAA-3′
ATP1A1 REV	5′-TCCACCTTGCAGCCATTT-G-3′
ENO2 FW	5′-TGA-CAA-GGC-TGG-CTA-CAC-AGA-3′
ENO2 REV	5′-CAT-ATT-TGC-CAT-CGC-GGT-AA-3′
ZO1 FW	5′-TTC GAT TGG CCA GCCATA TAT-3′
ZO1 REV	5′-TGT TTTCCGTCA CGG TAC CA-3′
NCAM1FW	5′-CGGATCTCGGTGGTATGGAA-3′
NCAM1REV	5′-CCGCAGTGACCACACACTTG-3′
SLC4A4FW	5′-GTGCTTGTTGGCGAGGTAGAC-3′
SLC4A4 REV	5′-GGACTTGGCTTTCCCCTTAGG-3′
CDH2 FW	5′-AGCAGTAAAACTGAGCCTCAAACC-3′
CDH2 REV	5′-TGC CTC TGC AGG TAG CCA TT-3′
GAPDH FW	5′-AGGGTCATCATCTCTGCACCTT-3′
GAPDH REV	5′-TGGTCATAAGTCCCTCCAACGG-3′

## Data Availability

The data presented in this study are available on request from the corresponding author.
